# The Tumour Microenvironment and Epigenetic Regulation in *BRCA1* Pathogenic Variant-Associated Breast Cancers

**DOI:** 10.3390/cancers16233910

**Published:** 2024-11-21

**Authors:** Jun Yu Tay, Josh Xingchong Ho, Fan Foon Cheo, Jabed Iqbal

**Affiliations:** 1 Lee Kong Chian School of Medicine, Imperial College London-Nanyang Technological University, Singapore 308232, Singapore; 2 Department of Anatomical Pathology, Division of Pathology, Singapore General Hospital, Singapore 169856, Singapore

**Keywords:** hereditary breast cancer, *BRCA1* and *BRCA2* pathogenic variants, tumour microenvironment, epigenetics, lysine-specific demethylase

## Abstract

**Background/Objectives**: *BRCA1* pathogenic variant (PV)-associated breast cancers are most commonly seen in hereditary genetic conditions such as the autosomal-dominant Hereditary Breast and Ovarian Cancer (HBOC) syndrome, and rarely in sporadic breast cancer. Such breast cancers tend to exhibit greater aggressiveness and poorer prognoses due to the influence of *BRCA1* pathogenic variants (PVs) on the tumour microenvironment. Additionally, while the genetic basis of *BRCA1* PV breast cancer is well-studied, the role of epigenetic mediators in the tumourigenesis of these hereditary breast cancers is also worth exploring. **Results**: PVs in the *BRCA1* gene interact with stromal cells and immune cells, promoting epithelial–mesenchymal transition, angiogenesis, and affecting oestrogen levels. Additionally, *BRCA1* PVs contribute to breast cancer development through epigenetic effects on cells, including DNA methylation and histone acetylation, leading to the suppression of proto-oncogenes and dysregulation of cytokines. In terms of epigenetics, lysine-specific demethylase 1 (LSD-1) is considered a master epigenetic regulator, governing both transcriptional repression and activation. It exerts epigenetic control over *BRCA1* and, to a lesser extent, *BRCA2* genes. The upregulation of LSD-1 is generally associated with a poorer prognosis in cancer patients. In the context of breast cancer in *BRCA1/2* PV carriers, LSD-1 contributes to tumour development through various mechanisms. These include the maintenance of a hypoxic environment and direct suppression of *BRCA1* gene expression. **Conclusions**: While LSD-1 itself does not directly cause mutations in *BRCA1* or *BRCA2* genes, its epigenetic influence sheds light on the potential role of LSD-1 inhibitors as a therapeutic approach in managing breast cancer, particularly in individuals with *BRCA1/2* PVs. Targeting LSD-1 may help counteract its detrimental effects and provide a promising avenue for therapy in this specific subgroup of breast cancer.

## Simple Summary

This review article sheds light on the differences in tumour microenvironment between sporadic breast cancers and *BRCA1* and, to a lesser extent, *BRCA2* pathogenic variant (PV)-associated breast cancers, which are usually hereditary. It also uncovers the epigenetic regulation behind the tumourigenesis and differences in tumour microenvironment, mainly exploring the role of the master epigenetic regulator, lysine-specific demethylase 1 (LSD-1). Finally, it provides a gist of current therapeutics in the domain of hereditary breast cancer and includes recommendations for areas of further research into the topic.

## 1. Introduction

*BRCA1* and *BRCA2* are tumour suppressor genes responsible for repairing double-strand DNA breaks via the homologous recombination repair (HRR) pathway. While there are many human *BRCA* variants, only a small proportion are pathogenic variants (PVs). These PVs are expressed in an autosomal dominant pattern with incomplete penetrance. Population-based studies put its penetrance for breast cancer at around 45% [1]. Roughly 10% of breast cancers result from inherited PVs in the *BRCA1* and *BRCA2* genes [2], most of which can be inherited in an autosomal dominant fashion as part of the Hereditary Breast and Ovarian Cancer (HBOC) syndrome [3] and greatly increase the risk of developing breast cancer. It is estimated that female carriers of *BRCA1/2* PVs are at a 69–72% risk of developing breast cancer by 80 years of age [4]. In women diagnosed with HBOC syndrome, the risk of the contralateral breast developing cancer is also significantly higher.

Hereditary breast cancer refers to a pluralistic group of genes that, when inherited, greatly increase the risks of breast cancer. The most common genes implicated are the *BRCA1* and *BRCA2* genes, and less common genes such as *PALB2*, *CHEK2,* and *PTEN* can rarely also be involved [5,6]. In particular, *BRCA1* PVs have been found to have unique effects on the tumour microenvironment (TME) compared to other sporadic breast cancers. These differences are likely contributory factors for the increased aggressiveness associated with *BRCA1* PV-associated breast cancers [7,8].

While the genetic basis of *BRCA1/2* hereditary breast cancer is well-studied, the role of epigenetic mediators in the tumourigenesis of these hereditary breast cancers is also worth exploring because the expression and suppression of these genes do have a component of epigenetic regulation [9]. Epigenetics refers to the study of changes in gene function that do not involve a change in DNA sequence or chromosomal aberrations. These modifications may even be inherited mitotically or meiotically [10]. A key player in such epigenetic dysregulation is lysine-specific demethylase 1 (LSD-1) because of its high levels of expression in hormone-negative breast cancer as well as its role in the tumour microenvironment of hereditary breast cancer [11].

### Objectives and Overview

This article has three main objectives. Firstly, it conducts a literature review and investigates the molecular mechanisms that contribute to the differences in the tumour microenvironment between *BRCA1* PV-associated breast cancers and other sporadic breast cancers. Specifically, it will explore the role of LSD-1 and other epigenetic mechanisms that sustain these tumour microenvironments. Secondly, it will examine the clinical implications of the interactions between *BRCA1*, and to a lesser extent *BRCA2* PVs, the tumour microenvironment, and epigenetic regulation in terms of prognosis and treatment for hereditary breast cancer. Finally, the article will provide recommendations for further research to address gaps in our current understanding of the epigenetic regulation of hereditary breast cancer. A glossary of the abbreviations and acronyms used in this article can be found in Table S1 as a Supplementary Materials.

## 2. The Impact of *BRCA1/2* PVs on the Tumour Microenvironment

### 2.1. Epithelial to Mesenchymal Transition

*BRCA1* PVs have been shown to alter the TME by directly enhancing epithelial-to-mesenchymal transition (EMT) in tumour cells. Physiologically, the EMT process plays a crucial role in embryogenesis and wound healing. During this process, the epithelial cells lose their polarity and intercellular adhesions but acquire proteins found in mesenchymal cells, which facilitates travel to the stromal microenvironment.

EMT is implicated pathogenically in tumourigenesis as well, with the loss of E-cadherin being the key process. One factor that can repress the transcription of E-cadherin and thus promote cancer is the TWIST protein. It has been shown that *BRCA1* binds to the TWIST promoter, suppressing its activity and inhibiting the EMT process [12]. Thus, *BRCA1* PVs result in TWIST overexpression and tumourigenesis. Slug (protein product of SNAI2) is another such factor that can repress the transcription of E-cadherin, and it has been reported to be upregulated in the presence of *BRCA1* PVs, despite *BRCA1* not being a transcriptional repressor of it [13,14]. *BRCA1* PVs have also been thought to induce aberrant interaction of breast cancer cells with other cell surface and cytoskeletal proteins responsible for the regulation of EMT such as P-cadherin, beta-catenin, vimentin, and cytokeratins [15].

Ultimately, *BRCA1* PVs can trigger EMT in luminal stem cells and induce transdifferentiation. By stimulating the proliferation of stem cells, it multiplies the risk of carcinogenesis, including the risk of developing into basal-like tumours [16,17,18,19], a subtype that portends a worse prognosis.

### 2.2. Stromal Cells

*BRCA1* PVs also influence the tumour microenvironment through its effects on surrounding stromal cells. In sporadically occurring breast cancer, mesenchymal cells have been shown to promote EMT [20,21], but this enhancement of EMT by mesenchymal stromal cells has been shown to be upregulated by *BRCA1* PVs, thereby further increasing the metastatic potential of tumours carrying *BRCA1* PVs [22,23,24].

**Figure 1:**
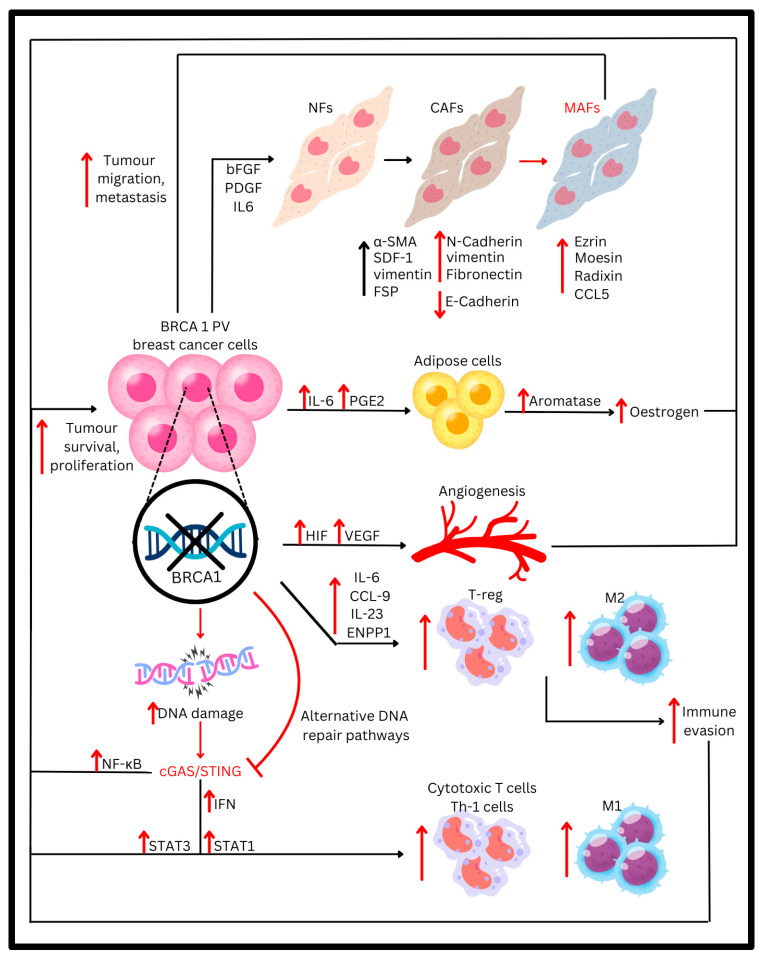
A simplified overview of how *BRCA1* PVs influence the tumour microenvironment to enhance tumour aggressiveness. Arrows, proteins, and cells marked in black are common to both sporadic breast cancer and *BRCA1* PV tumours, while changes marked in red are specific to *BRCA1* PV tumours.

Apart from mesenchymal stromal cells, *BRCA1* PVs also can influence cancer-associated fibroblasts (CAFs) to further promote metastasis of cancer cells. CAFs are dysfunctional fibroblasts closely associated with tumourigenesis. In sporadic breast cancers, tumour cell secretion of cytokines, such as IL-6, basic fibroblast growth factor, and platelet-derived growth factor (PDGF) α/β, transforms normal fibroblasts (NFs) into CAFs. These CAFs upregulate biomarkers such as alpha-smooth muscle actin (α-SMA), vimentin, fibroblast surface protein, and stromal-derived factor 1 [25] (Figure 1). Through secreting enzymes and cytokines altering the extracellular matrix such as vascular endothelial growth factors (VEGFs) and matrix metalloproteinases [26], CAFs frequently promote tumour progression by enhancing angiogenesis, growth, and invasion of the tumour (Table 1).

However, in the presence of *BRCA1* PVs, CAFs reduced the expression of E-cadherin while overexpressing fibronectin, vimentin, and N-cadherin, allowing for easier induction of EMT of tumour cells [27]. CAFs were also able to transform into metastasis-associated fibroblasts (MAFs) and increase their expression of EMT markers such as Ezrin, Radixin Moesin and CCL5 (Figure 1), which are important for tumour cell mobility, to further induce metastatic changes in breast cancer cells and augment tumour proliferation, migration, and invasion (Table 1) [27].

### 2.3. Oestrogen

Breast cancer cells harbouring *BRCA1* PVs contribute to elevated levels of local oestrogen within the tumour microenvironment, leading to increased proliferation and growth of oestrogen-dependent tumours. Breast cancer cells stimulate surrounding adipose stromal cells to produce aromatase, an enzyme responsible for oestrogen synthesis, by releasing factors such as IL-6 and Prostaglandin-E2. Normally, *BRCA1* suppresses the expression of the aromatase gene in stromal cells. However, *BRCA1* PVs result in excessive production of oestrogen [23] (Figure 1). Oestrogen, in turn, can directly induce genomic rearrangements that contribute to tumourigenesis. Although tumours carrying *BRCA1* PVs typically do not express oestrogen receptor alpha (ERα), studies have demonstrated that these cells can still respond to elevated oestrogen levels independently of oestrogen receptor expression [29].

Conversely, most sporadic breast cancers rely on oestrogen receptors (ERs) that are found in ER-positive breast cancer subtypes for oestrogen to act on [28] (Table 1).

### 2.4. Angiogenesis

Pathogenic variants (PVs) in *BRCA1/2* genes can contribute to enhanced tumour angiogenesis. Angiogenesis plays a crucial role in the continuous growth of tumours and is regulated by both pro-angiogenic and anti-angiogenic factors. As cancer cells proliferate, their metabolic demands escalate, necessitating a more significant oxygen supply (Table 1). Consequently, rapidly growing tumours experience relative oxygen deficiencies, leading to the development of a hypoxic environment. In response, the activity of hypoxia-inducible factors (HIFs) is amplified. It has been shown that VEGF and HIF are more highly expressed in *BRCA1/2* PVs than sporadic breast cancers [30] (Figure 1), and this has been attributed to the fact that *BRCA1* can bind to the *VEGF* gene promoter, inhibiting its transcription and thus reducing VEGF secretion [31] (Table 1).

Apart from VEGF, it is postulated that *BRCA1* can affect other pro-angiogenic factors, particularly angiopoietin-1, by forming a repressive complex with C-terminal binding protein-interacting protein (CtIP) and zinc finger and *BRCA1*-interacting protein with KRAB domain-1 (ZBRK1), which then inhibits the expression of angiopoietin-1 [38].

### 2.5. Immune Response

Arguably the most important effect *BRCA1/2* PVs have on the tumour microenvironment is its influence on the immune response, as the association between the tumour microenvironment and inflammation is frequently complex and bidirectional.

In sporadic breast cancer, T-cells make up most of the lymphocytes in the tumour microenvironment [39]. Among them, CD8 T-cells, mediated by interferons (IFNs), serve to eliminate tumour cells, alongside natural killer (NK) cells. The CD8 T-cells are aided by Th1 cells, which are also mediated by IFNs and IL-12. Th1 cells recruit antigen-presenting cells for effective CD8 T-cell differentiation [32].

On the other hand, Tregs promote breast cancer progression through secretion of immunosuppressive cytokines like IL-10, transforming growth factor beta (TGF-β), as well as direct cell–cell contact suppression [33,34].

Macrophages in the breast tumour microenvironment, called tumour-associated macrophages (TAMs), are similarly mediated by these cytokines. M1 macrophages, which exert anti-tumourigenic effects, are stimulated by IFN and tumour necrosis factor-alpha (TNF-α), while M2 macrophages, which have the opposite effect, are activated by IL-4, IL-10, and IL-13. Dendritic cells and neutrophils also behave likewise, with myeloid dendritic cells and N1 neutrophils having an anti-tumourigenic effect, and plasmacytoid dendritic cells and N2 neutrophils having a pro-tumourigenic effect [35,36] (Table 1). These leukocytes not only affect breast cancer development by regulating CD8 T-cells, NK cells, and regulatory T cells, but also through their effects on other components of the breast tumour microenvironment. For example, macrophages have been found to secrete cytokines, which regulate angiogenesis [40].

The tumour microenvironment can conversely affect the pro or anti-tumorigenicity of this inflammation. Studies have found polarisation and infiltration of leukocytes can be influenced by other cells in the tumour microenvironment, such as CAFs, which notably induce pro-tumourigenic polarisation of macrophages, and promote monocyte recruitment [41,42].

This inflammatory signalling in breast cancers has been found to be augmented in the presence of *BRCA1/2* PVs, as evidenced by the higher rates of lymphocytic infiltration as compared to sporadic breast cancers generally [8]. This is in part due to the increased genomic instability and tumour mutational burden, leading to unresolved DNA lesions more frequently occurring. As virtually all *BRCA1/2* PV breast cancers also harbour inactivating *TP53* mutations [43], such DNA lesions are also frequently carried into mitosis, resulting in the formation of micronuclei [44,45,46].

These micronuclei are mis-segregated chromosomes surrounded by a single lipid bilayer not part of the main nucleus, which can rupture and release DNA into the cytoplasm [46]. The cell responds to such ‘self’ DNA in the cytoplasm similarly to how it would to microbial DNA, with the help of cytosolic DNA sensor cyclic GMP-AMP synthase (cGAS) as part of the cell’s innate immune response [47,48,49]. cGAS activation catalyses the production of cyclic 2′3′ GMP-AMP (cGAMP), which triggers stimulation of interferon gene (STING)-dependent inflammatory signalling. This process results in the release of cytokines, especially IFNs [50], which support the proliferation of CD8 toxic T cells and Th-1 cells [51] and thus have an anti-tumourigenic effect (Figure 1).

While the activation of cGAS/STING may seem detrimental to tumour proliferation at first, cells carrying pathogenic variants (PVs) of *BRCA1/2* genes simultaneously possess many mechanisms to suppress cGAS signaling and evade immune clearance, and hence they also possess greater immune evasion compared to sporadic breast cancers. One of the primary strategies employed is the prevention of cytoplasmic DNA generation. This is accomplished by utilising alternative, non-conservative DNA repair pathways to mend double-strand breaks. Notably, cancer cells harbouring *BRCA1/2* PVs commonly rely on alternative repair pathways such as polymerase θ (POLQ)-mediated alternative end-joining and radiation sensitive 52 (RAD52)-mediated single-strand annealing [52]. Studies have shown that POLQ is upregulated in HR-deficient cancers, including those with *BRCA1/2* PVs, and inhibiting POLQ leads to micronuclei formation and IFN signaling [53]. Additionally, cancerous inhibitors of protein phosphatase 2A (Cip2A) and topoisomerase II-binding protein 1 (TopBp1) have been implicated in *BRCA1/2* PV cells. They form a complex with a mediator of DNA damage checkpoint 1 (Mdc1) to anchor chromosome fragments during mitosis, thereby preventing micronuclei generation [54,55].

Another possible way *BRCA1/2* PV cells clear cytoplasmic nucleic acids is through the utilisation of three prime repair exonuclease 1 (TREX1) enzymes and RNaseH1, which degrades cytoplasmic DNA [56,57,58]. However, their significance as a compensatory response is unclear as of now.

Additionally, other studies have shown that cGAS/STING may not entirely be an impediment to tumour growth. The JAK/STAT pathway activated by IFNs has growth-suppressing and proapoptotic effects if STAT1 is activated, but should STAT3 be activated instead, proliferation and prevention of apoptosis of tumour cells would conversely occur [59]. Additionally, STING also activates NF-κB through both its canonical and non-canonical pathway, which can lead to anti-tumourigenic effects, such as through induction of TAM repolarisation towards the M1 phenotype, but can also result in pro-tumourigenic effects, such as through upregulation of anti-apoptotic genes and promotion of cell survival [60,61] (Figure 1).

Activating mutations of oncogenes such as *C-MYC* found in *BRCA1/2* PV breast cancers [62] further blunt the immune response in the tumour microenvironment. Various immunosuppressive cytokines have been found to be upregulated in *BRCA1/2* PV cells, such as IL-10, as well as CCL-9 and IL-23, due to the increased expression of *C-MYC* [63], which suppresses IFN signalling and thus the activation of pro-inflammatory cytokines [64,65,66].

Some studies have also found *BRCA1* PVs to be associated with higher levels of PD-L1/PD-1 expression [67], while others have found certain *TP53* mutations cause binding of p53 to TBK1, inactivating the STING/TBK1/IRF3 pathway and furthering the immunosuppressive state of the tumour microenvironment [68].

Interestingly, in chromosomally unstable cancers like those containing *BRCA1/2* PVs, ectonucleotide pyrophosphatase/phosphodiesterase 1 (ENPP1) has been shown to be upregulated [69]. ENPP1 not only hydrolyses cGAMP, but it also breaks down extracellular adenosine triphosphate (ATP), resulting in the formation of AMP, which is then broken down further by 5′-Nucleotidase Ecto (NT5E) to form adenosine [69], which is immunosuppressive within the TME. This likely also contributes to the increased infiltration of regulatory T cells within the TME seen in *BRCA1/2* PV cancers [37,70]. A summary of differences in immune regulation between *BRCA1/2* PV and sporadic breast cancers can be found in Table 2.

However, it is important to note that *BRCA1/2* PV and sporadic breast cancers have a wide range of phenotypes which are not mutually exclusive. Though *BRCA1/2* PV breast cancers generally have greater immune infiltration [8] and simultaneously a more immunosuppressive microenvironment than sporadic breast cancers, sporadic breast cancers with high degrees of homologous repair deficiency can present phenotypically similarly to *BRCA1/2* PV breast cancers with increased numbers of infiltrating lymphocytes [71], as well as decreased immune cytotoxicity and thus higher degrees of immune evasion [72].

## 3. Epigenetic Modification Mechanisms in Hereditary Breast Cancer

**Figure 2:**
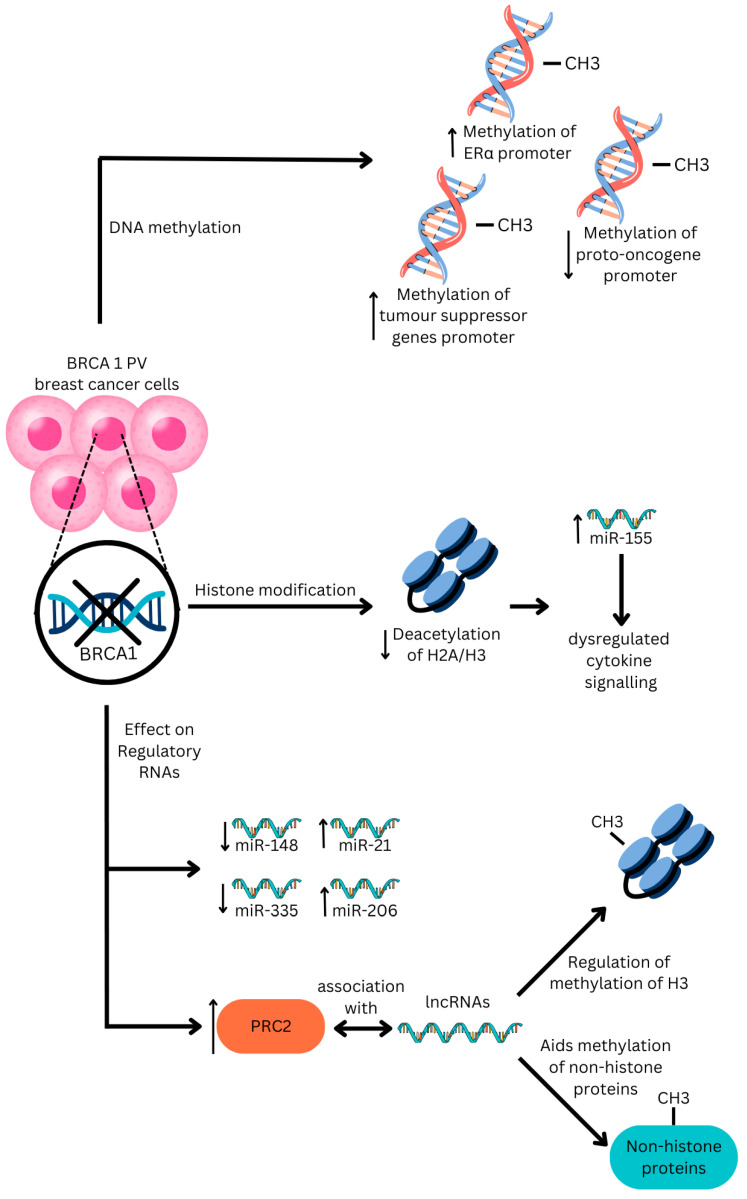
An overview of the mechanisms of epigenetic modification by *BRCA1* PVs, illustrating how DNA methylation, histone modification, and regulatory non-coding RNAs are influenced by *BRCA1* PVs to result in increased tumour aggressiveness. In *BRCA1* PV-associated breast cancer, the promoter region of ERα is more highly methylated, while deacetylation of histones H2A and H3 are impaired. In the figure, upward pointing arrows (↑) refer to upregulation while downward pointing arrows (↓) refer to downregulation.

While *BRCA1/2* PVs have an extensive influence on tumourigenesis by affecting the tumour microenvironment, the differences in epigenetics of hereditary and sporadic breast cancers also predispose hereditary *BRCA1* PV carriers and, to a lesser extent, *BRCA2* ones, to the development of cancer. They do so by contributing to and perpetuating the tumour microenvironment of hereditary breast cancer. Generally, three classes of epigenetic regulation exist to regulate gene expression [10,73,74]. A summary of the epigenetic mechanisms can be found in Figure 2.

### 3.1. DNA Methylation

DNA methylation takes place primarily in cytosine nucleotide bases within CpG dinucleotide sequences which are more often found in the promoter regions of silenced genes than ones with active transcription [75,76]. Methylated cytosine can then block the binding of transcription factors and recruit other repressors of transcription including histone deacetylases that lead to chromatin remodelling. However, DNA methylation has also been found to take place across non-CpG sequences [10]. The mechanism of DNA demethylation, on the other hand, is less understood than that of methylation, although it has been implicated in a variety of conditions such as cardiovascular diseases and malignancies [74].

In *BRCA1* PV breast cancers, the promotor region of the ERα is highly methylated compared to the sporadic breast cancers [77,78]. Such a phenomenon provides a possible explanation for the low expression of ERα in hereditary breast cancers. In both normal breast tissue and in breast cancer, methylation levels of the promoter regions of tumour suppressor genes such as *BRCA1*, *BRCA2,* and *ESR1* were higher in both *BRCA1/2* PV carriers than non-carriers [77,79,80].

Various studies have also shown that there is a global DNA hypomethylation in tumour cells with respect to sporadic breast cancers [78,81,82]. The hypomethylation of proto-oncogenes increases the expression of these genes, hence facilitating tumourigenesis. Examples of proto-oncogenes that have been shown to be upregulated include *RAD9*, a gene implicated in cell cycle control and DNA repair [81].

### 3.2. Histone Modification

Histones can undergo a plethora of post-translational epigenetic modifications such as acetylation or deacetylation, methylation or demethylation, and phosphorylation or dephosphorylation that consequently alter gene expression. The best-studied histone modifications are the acetylation and methylation of lysine residues on histones H3 and H4. Notably, unlike histone acetylation and deacetylation, which promote and repress transcription, respectively, the effects of histone methylation and demethylation are more ambiguous, depending on the position of the lysine residue and extent of methylation [10,74,75,76].

In most sporadic breast cancers with intact *BRCA1* and *BRCA2* genes, the C-terminal domain of the *BRCA* protein interacts with histone deacetylases to promote the deacetylation of histones as well as other genes [80,82]. For instance, HDAC1 complexes with the *BRCA1* protein to deacetylate genes involved in the non-homologous recombination pathway of DNA repair while HDAC2 complexes with the *BRCA1* protein to deacetylate histones H2A and H3 [83]. In hereditary *BRCA1/2* PV breast cancers, the knockout of *BRCA* proteins results in impaired histone deacetylation [84]. An example of the impact of a lack of deacetylation of histones H2A and H3 would be the upregulation of the miR-155 promoter and the overexpression of micro-RNA 155 (miR-155), which leads to a dysregulation in cytokine signalling pathways as well as the facilitation of EMT [84].

### 3.3. Regulatory Non-Coding RNA Action

Non-coding RNAs such as small inhibitory RNAs (siRNAs) and microRNAs (miRNAs) repress transcription by promoting DNA methylation and histone modifications mediated by proteins such as argonaute. The mechanisms behind which these regulatory non-coding RNAs regulate gene expression remain to be elucidated [10,85,86,87,88].

Long non-coding RNAs (lncRNAs), including circular RNAs (circRNAs), have also been shown to be part of chromatin-modifying complexes that when downregulated reverse the silencing of genes by the polycomb repressive protein complex 2 (PRC2) [89]. They are also involved in the regulation of methylation of lysine 4 and lysine 9 of histone H3 (H3K4 and H3K9, respectively) by interacting with trithorax group (TrxG) proteins [90]. Moreover, lncRNAs also have a role in the methylation of non-histone proteins such as beta-catenin by acting as scaffolding for methyltransferases and other transcriptional enzymes [91,92].

Studies have shown that in *BRCA1/2* PV hereditary breast cancer, certain miRNAs such as miR-148 and miR-335 were downregulated, while certain ones were upregulated, such as miR-21 and miR-206, although the correlation between the varying quantities of non-coding RNAs and differences in tumourigenesis was found to be weak [77]. Likewise, while PRC2 is upregulated in *BRCA1/2* PV hereditary breast cancer [10], the specificity of its association with lncRNAs remains elusive due to their diverse derivations [89], although various lncRNAs such as HOX antisense intergenic RNA (HOTAIR) and promotor of CDKN1A antisense DNA damage-activated RNA (PANDAR) have shown to play a role in breast cancer tumourigenesis through various mechanisms [93].

## 4. The Role of LSD-1 and Other Enzymes in the Epigenetic Regulation of Hereditary Breast Cancer

### 4.1. The Significance of LSD-1

LSD-1 is a prototypical histone demethylase enzyme involved in epigenetic processes that has been implicated in the pathogenesis of breast cancer as well as many other tumours [10,74,75,76]. It has also been associated with a poor cancer prognosis [94,95,96,97]. In terms of enzymatic activity, LSD-1 catalyses the demethylation of mono-methylated or di-methylated lysine 4 on histone H3 (H3K4me1 and H3K4me2, respectively). However, depending on the substrate, LSD-1 has been shown to have epigenetic effects on both transcriptional activation [75,76] as well as repression [74,98,99,100].

Besides this, LSD-1 can also demethylate non-histone proteins such as tumour suppressor proteins to effect epigenetic influences. Examples include the demethylation of K370 lysine residue of *p53*, lysine 442 of *MYPT1,* which is an important regulator of the dephosphorylation of the retinoblastoma protein (*pRb1*), as well as lysine 185 of the *E2F1* transcription factor [75,76]. These all work to suppress the expression and effects of tumour suppressor proteins.

Due to its versatility as an epigenetic modulator, LSD-1 has been touted to be a master regulator controlling cellular homeostasis [10]. As such, it has a complementary role alongside *BRCA1/2* PVs and is inextricably intertwined with cellular processes that contribute to tumourigenesis in hereditary breast cancer.

### 4.2. LSD-1 and the Tumour Microenvironment in General Cancer Pathogenesis

The role of LSD-1 in EMT is evident from the global H3K9me2 reduction during the transition process. By binding to the SNAI-1 protein which, together with the Slug protein, represses E-cadherin, LSD-1 contributes to the loss of cellular adhesions between cancer cells and augments their ability to invade and metastasise [75].

In addition, by catalysing the demethylation of tumour suppressor proteins such as p53 and E2F1, LSD-1 downregulates the expression of these non-histone proteins [101,102].

Moreover, LSD-1 regulates hypoxia through the demethylation of HIF-1α to stabilise it [10] and allow tumour cells to proliferate without the consumption of oxygen during respiration [75]. LSD-1 also indirectly contributes to the stability of HIF-α through a series of interactions with other proteins such as the demethylation of the RACK-1 and the inhibition of HIF-1α hydroxylation that mediates its degradation [10,103].

Lastly, raised LSD-1 levels have been correlated with a shift from mitochondrial to glycolytic respiration, which is a hallmark of most cancer cells. Through the demethylation of genes such as acyl-CoA dehydrogenase medium chain (ACADM), LSD-1 represses mitochondrial respiration. Conversely, decreasing LSD-1 levels is associated with a decrease in glucose uptake and glycolysis, consequently activating mitochondrial respiration [104].

### 4.3. The Association with Aggressive Subtypes of Breast Cancer

Of the 4 molecular subtypes of breast cancer—basal-like, luminal A, luminal B, and HER2 positive—the type most strongly associated with LSD-1 overexpression is basal-like breast cancer, which, as previously mentioned, is also more likely to occur in individuals carrying *BRCA1* PV. Basal-like breast cancers frequently do not express hormonal receptors and HER2, with many basal-like breast cancers being triple-negative breast cancers (TNBC) and vice versa. These cancers have the worst prognosis, with many patients being of a younger age and having a larger tumour size on diagnosis [105]. Not only is the overexpression of LSD-1 linked to more aggressive subtypes of breast cancer, but it is also associated with poorer outcomes in these subtypes of breast cancer, such as shorter recurrence-free survival and higher hazard ratios for recurrence [97].

This is in stark contrast to sporadic breast cancers, which are predominantly of the luminal A breast subtype harbouring the best prognosis [97].

### 4.4. Downregulation of BRCA1 and BRCA2

The overexpression of LSD-1 in breast cancer has been correlated with a downregulation of *BRCA1*, especially in aggressive cancers such as basal-like and TNBC [10]. This is because, in these cancers, the Wnt signalling is upregulated, leading to an upregulation of the expression of the transcription repressor Slug together with an accumulation of *β*-catenin. The SNAG domain on Slug interacts with LSD-1, forming a complex that binds to the promoter region of *BRCA1* and represses its expression [106].

The effects of downregulating *BRCA1* by LSD-1 are arguably more pronounced in hereditary breast cancer with *BRCA1* PV because of the additional component of genetic instability, fewer functional *BRCA* proteins, and an increased likelihood of loss of heterozygosity, in which the wild-type alleles of the *BRCA* genes are lost [106,107,108].

The perpetuation of a hypoxic tumour microenvironment by LSD-1 also contributes to the downregulation of *BRCA2* [109]. Observational studies have also shown that levels of expression of either *BRCA* gene were closely linked to the other, and that women with *BRCA1/2* PV have similar or overlapping regulatory pathways [110], the mechanism of which remains to be investigated. Once again, the effects of downregulating functioning *BRCA2* gene copies are more pronounced in patients with *BRCA2* PVs.

### 4.5. Enhancer of Zeste Homologue 2 and Other Enzymes Involved in Hereditary Breast Cancer Epigenetic Regulation

Other noteworthy enzymes involved in the perpetration of a conducive tumour microenvironment by means of epigenetic regulation in hereditary breast cancer include histone methyltransferases such as the enhancer of zeste homologue 2 (EZH2) that represses target gene expression [9] and lysine methyltransferase 2 (KMT2) that promotes the expression of oestrogen-dependent oncogenes like the epidermal growth factor [111].

EZH2 is a subunit of the PRC2 which tri-methylates lysine 27 of histone H3 (H3K27me3) to downregulate its expression [112,113]. Similar to LSD-1, it has been observed to be upregulated in hereditary breast cancer and has been proposed to be a biomarker for aggressive breast cancer [10]. Independent of PRC2, EZH2 regulates the shuttling of *BRCA1* protein from the nucleus to the cytoplasm in basal-like breast cancer cells. By reducing the nuclear localisation of phospho-BRCA1 and increasing the expression of phosphor-Akt1, it increases nuclear retention of *BRCA1* proteins, thereby contributing to tumourigenesis by promoting aberrant mitosis, aneuploidy, and ultimately genomic instability [112].

## 5. Therapeutics in Hereditary Breast Cancer

The management of hereditary breast cancers is different from that of sporadic ones [6]. There are higher rates of mastectomies as well as chemotherapy-only adjuvant and neoadjuvant regimes in *BRCA1/2* PV-related breast cancers than in sporadic breast cancers. On the other hand, the chances of hereditary breast cancer patients receiving hormone therapy without chemotherapy are lower [114]. This is because *BRCA1* PV-associated breast cancers, most of which are hereditary, predispose patients to triple-negative and basal-like cancers. The differences in methylation status also affect the responsiveness of these cancers to immunotherapy [10]. Specifically, poly ADP-ribose polymerase (PARP) inhibitors such as olaparib have been shown to be an effective adjunct therapy as part of the OlympiA trial to improve survival outcomes in *BRCA1/2* hereditary breast cancers [115,116], although recent studies on their use in sporadic breast cancers having somatic *BRCA* mutations have also yielded promising outcomes, albeit rare [117].

### 5.1. LSD-1 Inhibitors and Their Current Trials and Applications

Due to LSD-1 being implicated in several cancers, LSD-1 inhibitors, many of which are derived from monoamine oxidase (MAO) inhibitors owing to their structural similarity, have been developed as a therapeutic modality [74,75,76]. One of the first such inhibitors to be identified was tranylcypromine (TCP), an irreversible inhibitor of LSD-1. Others include the reversible inhibitors GSK354 and GSK2879552 [10]. Moreover, natural bioactive compounds such as flavones, xanthones, and melatonin have all been found to have LSD-1-inhibiting properties and also offer promising results in the development of new LSD-1 inhibitors.

Chemical LSD-1 inhibitors have been successfully used to block the growth of embryonic stem cells, pluripotent carcinomas like teratomas and embryonic carcinoma, as well as leukaemia [76].

There have been clinical trials of LSD-1 inhibitors for other cancers, particularly for small-cell lung cancer (SCLC) and acute myeloid leukaemia (AML), demonstrating various potential uses and anti-tumour effects [118,119]. Results of selected trials are summarised in Table 3. These studies support the potential and prospects of the utility of LSD-1 inhibitors as targeted therapies in solid cancers like breast cancer.

### 5.2. LSD-1 Inhibitors in Breast Cancer Treatment

In terms of breast cancer, the LSD-1 inhibitor INCB059872 together with immunotherapy such as anti-programmed cell death ligand 1 drugs (anti-PD-L1) enhanced the efficacy of such immunotherapy agents and general anti-tumour efficacy [10]. Other studies have also found LSD-1 inhibition to increase the number of PD-L1 receptors on epithelial breast cancer cells and triple-negative breast cancer cells [120]. Given the lack of responsiveness of breast cancer to immunotherapy due to absence of a high tumour mutational burden and lymphocytic infiltration [10], the addition of LSD-1 inhibitors to the armamentarium of anti-tumour drugs represents a promising new therapy [121].

However, there have been relatively few clinical trials on the utility of LSD-1 inhibitors in the treatment of breast cancer [10]. For this, the European Union Clinical Trials Register (accessed on 24 October 2024) and the National Library of Medicine clinical trials registry (ClinicalTrials.gov) (assessed on 24 October 2024) were used as sources. An open-label Phase 1 trial by Prasanna et al. in 2022 has found that coupling phenelzine (an LSD-1 inhibitor) with Nab-Paclitaxel (a chemotherapy agent) for metastatic breast cancers has the potential to eliminate circulating tumour cells with aggressive mesenchymal phenotype [122]. There are however no ongoing trials investigating the interactions between LSD-1 and *BRCA1/2* for hereditary breast cancer.

### 5.3. LSD-1/NuRD Complexes and JQ1

Interestingly, despite its primarily oncogenic role, LSD-1 has also been found to contribute to tumour suppression through its association with the NuRD complex. As a component of the NuRD complex, LSD-1 inhibits genes involved in TGF-β signalling, thereby impeding EMT and suppressing cancer metastasis. Recent studies have highlighted the association of LSD-1/NuRD complexes with the suppression of luminal breast cancer metastasis [74]. Moreover, the suppression of pellino E3 ubiquitin protein ligase 1 (PELI-1), a destabiliser of the LSD-1/NuRD complex, which results in higher recruitment of LSD-1/NuRD complexes, has been shown to improve the prognosis of breast cancer as well as improve the efficacy of other therapeutics such as JQ1 in the treatment of both *BRCA1* PV-associated and non-*BRCA* breast cancer [123].

## 6. Conclusions and Discussion for Future Prospects

*BRCA1* PVs promote the formation of more aggressive breast cancers with worse prognoses through their influence on the TME. They are further associated with a preponderance to the development of other cancers such as cancer of the contralateral breast and ovarian cancer [124,125], as well as pancreatic cancer and prostate cancer in males [126]. In a move to go above and beyond genetic predisposition, this review has attempted to draw a comprehensive comparative analysis between the differences in tumour microenvironment and differences in epigenetic regulation which result in differences in methylation and acetylation states in *BRCA1* PV-associated cancer compared to sporadic breast cancer with intact *BRCA1* proteins. These differences all contribute to the more aggressive tumourigenesis of *BRCA1* PV-associated cancer. With LSD-1 being revealed as a potent epigenetic regulator of *BRCA1/2* and thus in breast cancer tumourigenesis, it could prove to be a key target in hereditary breast cancer treatment.

While it is postulated that the role of LSD-1 in epigenetic regulation differs across cancer-specific contexts [127], the interplay between the promoting and inhibiting roles of LSD-1 in EMT and the mechanisms that reconcile these processes remain to be fully understood, offering a promising area for future research.

The focus of research on *BRCA1* PVs has overshadowed investigations into *BRCA2* and its relationship with the TME and LSD-1. Exploring how LSD-1 downregulates *BRCA2* and its impact on the TME could unveil new avenues for the treatment of hereditary breast cancer.

Considering the ongoing trials of LSD-1 inhibitors in breast cancer treatment, it is worthwhile to consider their potential as prophylactic therapy. Currently, women who carry *BRCA1/2* PVs but do not have breast cancer are offered risk management options such as intensified risk surveillance, risk-reducing bilateral mastectomy (RRBM), and chemoprevention. Chemoprevention, typically through selective oestrogen receptor modulators like Tamoxifen, is employed on a case-by-case basis. The use of LSD-1 inhibition as an epigenetic intervention could present an alternative approach, potentially eliminating the need for surgery or long-term medication use.

## Figures and Tables

**Table 1:** A summary of qualitative and quantitative differences in the cells and proteins in the tumour microenvironment of sporadic breast cancers and *BRCA1* PV tumours.

	SPORADIC BREAST CANCER	*BRCA1/2* PV HEREDITARY BREAST CANCER
PATHOPHYSIOLOGY	Mutational activation of oncogenes through accumulation of stepwise mutations in somatic genes. *BRCA1/2* mutations are rare.	Germline mutation of one allele of *BRCA1/2*, followed by inactivation of the second allele. This results in increased genomic instability due to non-conservative repair of double-stranded DNA breaks.
STROMAL CELLS	Breast cancer cells induce transformation of normal fibroblasts (NFs) to CAFs through paracrine effects. Activated CAFs express classic biomarkers and secrete enzymes to enhance angiogenesis, growth, and tumour invasion [25,26].	CAFs reduce expression of E-cadherin and over-express fibronectin, vimentin and N-cadherin, which allows greater ease of EMT. CAFs can also transform into metastasis-associated fibroblasts (MAFs) which increase EMT markers to further induce metastatic changes [27].
OESTROGEN LEVELS	High oestrogen levels are a risk factor for sporadic breast cancer, causes of which are mostly not due to *BRCA1* mutations. Most breast cancers rely on oestrogen receptors (ERs) that are found in ER-positive breast cancer subtypes [28].	Oestrogen levels are elevated due to lack of suppression by *BRCA1* protein, which stimulate surrounding adipose stromal cells to produce aromatase [23]. Oestrogen in turn can directly induce genomic rearrangements that contribute to tumourigenesis [29]. *BRCA1* PV tumours can also respond to elevated oestrogen levels independently of oestrogen receptor expression [29].
ANGIOGENESIS	Increased metabolic demands result in relative oxygen deficiency, leading to upregulation of HIFs.	HIFs and VEGFs are even more highly expressed compared to sporadic breast cancers. *BRCA1* has been postulated to play a role in HIF and VEGF inhibition, as well as inhibition of other pro-angiogenic factors thus *BRCA1* mutation results in disinhibition of these factors [30,31].
IMMUNE RESPONSE	CD8 T-cells, NK cells, Th1 cells, M1 macrophages, N1 neutrophils, and myeloid dendritic cells, aided by Th1 cytokines have anti-tumourigenic effects. Th2 cells, M2 macrophages, Tregs, N2 neutrophils, and plasmacytoid dendritic cells, aided by Th2 cytokines, promote breast cancer progression [32,33,34,35,36].	The higher degree of DNA damage induces greater immune cell signalling, resulting in greater numbers of immune cell infiltration, with higher numbers of T-cells and macrophages within the TME. However, the inflammatory response is also more pro-tumourigenic in nature with a greater proportion of immunosuppressive immune cells such as regulatory T-cells and M2 macrophages [37].

**Table 2:** A summary of how immune regulation in *BRCA1* PV breast cancers differs from that of sporadic breast cancer.

	*BRCA1/2* PV Breast Cancers Compared to Sporadic Breast Cancers			References
Increased immune cell infiltration	Micronuclei formation			[44,45,46]
	Increased cGAS/STING activation			[47,48,49]
	Increased NF-κB activation			[60,61]
	Increased IFN signalling			[50]
	Increased JAK/STAT1 activation			[59]
Greater Immunosuppression	Mitigation of micronuclei generation	Alternative repair pathways	RAD52	[52]
			POLQ	[53]
		Cip2A, TopBP1		[54,55]
	Decreased IFN signalling	C-MYC mutations		[63]
		Decreased STING/TBK1/IRF3 signalling from TP53 mutations		[68]
	Increased JAK/STAT3 activation			[59]
	Increased NF-κB activation			[60,61]
	Increased ENPP1			[69]
	Increased PD-L1/PD-1 expression			[67]
	Increased T-reg infiltration			[37,70]

**Table 3:** Trials utilising LSD-1 inhibitors as potential therapy options for other cancers and their findings. The trials were retrieved from American and European trial registries.

Trial Identifier	Drug	Cancer Type	Aims/Findings
NCT02913443	RO7051790	Solid (SCLC)	To determine the maximum tolerated and/or optimal dose for SCLC
EUDRACT 2013-002447-29	ORY-1001	Haematological (leukaemia)	ORY-1001 is well tolerated and promotes differentiation of blast cells
NCT02273102	Tranylcypr-omine	Haematological (AML/MDS)	TCP-ATRA combination, was well-tolerated with an acceptable safety profile
NCT05420636	Iadademstat	Solid (SCLC/G3 NEC)	To evaluate the efficacy of iadademstat-paclitaxel combination in refractory SCLC and Grade 3 neuroendocrine cancers

## Supplementary Materials

The following supporting information can be downloaded at https://www.mdpi.com/article/10.3390/cancers16233910/s1, Table S1: Glossary of abbreviations and acronyms.
