# Supplementary material for: The Tumour Microenvironment and Epigenetic Regulation in BRCA1 Pathogenic Variant-Associated Breast Cancers
Source: Cancers (Basel). 2024 Nov 21;16(23):3910. doi: 10.3390/cancers16233910 (PMC11639800; doi:10.3390/cancers16233910)
Supplement: Supplementary file 1 [file cancers-16-03910-s001.zip › cancers-3267255-supplementary.pdf]

Supplementary Materials: The Tumour Microenvironment and Epigenetic Regulation in *BRCA1* Pathogenic Variant-Associated Breast Cancers

Jun Yu Tay, Josh Xingchong Ho, Fan Foon Cheo and Jabed Iqbal

Table S1. Glossary of abbreviations and acronyms.

Abbreviation	Definition
ACADM	Acyl-CoA dehydrogenase medium chain
ATP	Adenosine triphosphate
CAF	Cancer associated fibroblasts
cGAMP	Cyclic 2′3′ GMP-AMP
cGAS	Cytosolic DNA sensor cyclic GMP-AMP synthase
Cip2A	Cancerous inhibitor of protein phosphatase 2A
CtIP	C-terminal binding protein-interacting protein
EMT	Epithelial to mesenchymal transition
ENPP1	Ectonucleotide pyrophosphatase/phosphodiesterase 1
ERα	Oestrogen receptor alpha
EZH2	Enhancer of zeste homologue 2
HOTAIR	HOX antisense intergenic RNA
HBOC	Hereditary Breast and Ovarian Cancer syndrome
HIF	Hypoxic inducible factors
HRR	Homologous recombination repair
IFN	Inteferon
KMT2	Lysine methyltransferase 2
LSD-1	Lysine-specific demethylase 1
MAF	Metastasis associated fibroblasts
MAO	Monoamine oxidase
Mdc1	Mediator of DNA damage checkpoint 1
miR-155	Micro-RNA 155
microRNAs	microRNAs
NT5E	5'-Nucleotidase Ecto
PANDAR	Promoter of CDKN1A antisense DNA damage-activated RNA
PARP	Poly (ADP-ribose) polymerase inhibitors
PD-L1	Programmed cell death ligand 1
PELI-1	Pellino E3 ubiquitin protein ligase 1
POLQ	Polymerase θ
pRb1	Retinoblastoma protein
PRC2	Polycomb repressive protein complex 2
PV	Pathogenic variant
RAD52	Radiation sensitive 52
RRBM	Risk-reducing bilateral mastectomy
siRNAs	Small inhibitory RNAs
SMA	Smooth muscle actin
STING	Stimulator of interferon genes
TAM	Tumour-associated macrophage
TCP	Tranylcypromine
TGF-β	Transforming growth factor beta

TME	Tumour microenvironment
TNBC	Triple-negative breast cancer
TNF-α	Tumour necrosis factor-alpha
TopBP1	Topoisomerase II-binding protein 1
TREX1	Three prime repair exonuclease 1
TrxG	Trithorax group proteins
VEGF	Vascular endothelial growth factors
ZBRK1	Zinc finger and *BRCA1*-interacting protein with KRAB domain-1
